# COVEVOL: Natural Evolution at 6 Months of COVID-19

**DOI:** 10.3390/v13112151

**Published:** 2021-10-25

**Authors:** Louise Messin, Marc Puyraveau, Yousri Benabdallah, Quentin Lepiller, Vincent Gendrin, Souheil Zayet, Timothée Klopfenstein, Lynda Toko, Alix Pierron, Pierre-Yves Royer

**Affiliations:** 1 Infectious Disease Department, Nord Franche-Comté Hospital, 90400 Trevenans, France; vincent.gendrin@hnfc.fr (V.G.); souheil.zayet@hnfc.fr (S.Z.); timothee.klopfenstein@hnfc.fr (T.K.); lynda.toko@hnfc.fr (L.T.); alix.pierron@hnfc.fr (A.P.); pierre-yves.royer@hnfc.fr (P.-Y.R.); 2 Clinical Investigation Center, Jean Minjoz University Hospital of Besançon, 25000 Besancon, France; mpuyraveau@chu-besancon.fr; 3 Department of Pneumology, Nord Franche-Comté Hospital, 90400 Trevenans, France; benabdallah.yosri@hotmail.fr; 4 Virology Department, Jean Minjoz University Hospital of Besançon, 25000 Besancon, France; q1lepiller@chu-besancon.fr

**Keywords:** post-COVID-19, “long COVID”, risk factors, persistent symptoms, asthenia, dyspnea, anxiety, anosmia

## Abstract

Many studies have investigated post-COVID symptoms, but the predictors of symptom persistence remain unknown. The objective was to describe the natural course of the disease at 6 months and to identify possible factors favoring the resurgence or persistence of these symptoms. COVEVOL is a retrospective observational descriptive study of 74 patients. All patients with positive SARS-CoV-2 PCR from March 2020 were included. We compared a group with symptom persistence (PS group) with another group without symptom persistence (no-PS group). Fifty-three out of seventy-four patients (71.62%) described at least one persistent symptom at 6 months of SARS-CoV-2 infection. In the PS group, 56.6% were women and the average age was 54.7 years old [21–89.2] ± 16.9. The main symptoms were asthenia (56.6%, *n* = 30), dyspnea (34%, *n* = 18), anxiety (32.1% *n* = 17), anosmia (24.5%, *n* = 13) and agueusia (15.1% *n* = 8). Ten patients (13.51%) presented a resurgence in symptoms. Patients in the PS group were older (*p* = 0.0048), had a higher BMI (*p* = 0.0071), and were more frequently hospitalized (*p* = 0.0359) compared to the no-PS group. Odynophagia and nasal obstruction were less present in the inaugural symptoms of COVID-19 in the PS group (*p* = 0.0202 and *p* = 0.0332). Persistent post-COVID syndromes are common and identification of contributing factors is necessary for understanding this phenomenon and appropriate management.

## 1. Introduction

In December 2019, a new coronavirus causing atypical pneumonitis, sometimes complicated by acute respiratory distress, was identified in Wuhan, China [1]. The global spread of severe acute respiratory coronavirus 2 (SARS-CoV-2) due to its high contagiousness is at the origin of an ongoing pandemic declared by the World Health Organization (WHO) in March 2020 [2]. The various symptoms caused by SARS-CoV-2 define the disease called COVID-19. These symptoms include: fever, myalgia, headache, asthenia, anosmia, agueusia and dyspnea. The two main life-threatening complications are acute respiratory distress syndrome (ARDS) and venous thromboembolic disease (VTED) [3]. Several risk factors for severity and mortality were identified: age over 65 years old, male gender, cardiovascular pathologies and their risk factors, chronic lung and kidney diseases and neoplasias [4]. In France, 5,993,937 cases have been confirmed with 111,644 deaths since 31 December 2019 [5]. Numerous international studies have described the clinical, epidemiological, pathophysiological, radiological and therapeutic features of COVID-19 in the acute phase. However, as COVID-19 is a new disease, few studies refer to the long-term clinical course and recovery of patients after SARS-CoV-2 infection. Some studies describe cases of COVID-19 recurrence of symptoms [6]. Many clinicians are confronted to the persistence of symptoms beyond 3 months for some of their patients. Similarly, in June 2020, the French National Academy of Medicine issued a statement calling on doctors to be vigilant with regard to patients recovering from COVID-19, given the persistence and resurgence of certain symptoms [7]. A study of 180 patients in the Faroe Islands showed that 53.1% of patients had at least one symptom after an average follow-up of 125 days after COVID-19 infection. These symptoms were mainly asthenia, loss of taste and smell, and arthralgias [8]. The term “long COVID” was used by patient associations before being used in the literature to describe this phenomenon. The Haute Autorité de Santé (HAS) prefers the broader term of prolonged symptoms following a COVID-19 infection [9]. It appears important to analyze the long-term natural behavior of this disease [10]. The main objective of this study was to describe the natural course of the disease at 6 months and to identify possible factors favoring resurgence or persistence of symptoms. 

## 2. Materials and Methods

We conducted a retrospective observational descriptive study of patients treated at the Nord Franche-Comté Hospital (NFCH). We included all major patients with a positive PCR for SARS-CoV-2 collected at NFCH in March 2020. Minors, patients who died at the time of collection and those who refused to participate in the study were excluded. Data related to each patient’s initial symptoms were retrospectively collected in March in a first study called COVIDES (retrospective observational study conducted from March 2020 in our establishment including all patients with positive SARS-CoV-2 PCR). As our patient sample was already included in COVIDES, we reused these initial retrospective data in patients eventually included in COVEVOL. The nonopposition to the use of the patients’ medical data was sought by sending them a letter mentioning the existence of COVIDES and COVEVOL. In the absence of a reply from them after a period of one month in accordance with the legislation in force, we retained their nonopposition.

The variables studied (apart from data already collected via COVIDES) were collected during a telephone interview, after patient consent, by a single operator using a standardized questionnaire of 18 questions between 13 July and 5 October 2020. These data have been anonymized. These variables concerned: demographic data, lifestyle, data related to the medical specificities, additional data concerning initial symptoms (asthenia, initial anxiety, duration of the first symptoms), data concerning the evolution of symptoms (presence of persistent symptoms, resurgence of symptoms), data concerning the results of complementary examinations at a distance from the infection (i.e., more than one month after the initial PCR: follow-up chest CT scan, 2nd SARS-CoV-2 PCR, SARS-CoV-2 serology), data related to the presence of persistent symptoms at 6 months (±2 months) of SARS-CoV-2 infection (dyspnea, asthenia, agueusia, anosmia, anxiety, other). Asthenia was measured according to the WHO performance status. Anxiety was measured using an ordinal scale (not anxious, slightly anxious, moderately anxious, highly anxious, very highly anxious). The intensity of dyspnea was measured using the mMRC (modified Medical Research Council) scale proposed by the French pneumology society. The degree of lung damage was estimated on chest CT scan, if applicable, according to the classification that was proposed by the French society of radiology for patients with suspected COVID-19 infection in March 2020. Patients with persistent dyspnea were seen in consultation to perform a clinical examination, oxygen saturation, blood gas, a 6-min walking test (6WT), lung functions tests (LFT) and low-dose chest CT scan. 

Detection of SARS-CoV-2 RNA was performed on flocked nasopharyngeal swabs (Eswab^®^, COPAN, Murrieta, CA, USA), nasopharyngeal aspirates, bronchoalveolar lavages or sputum transmitted to the virology laboratory of the Jean Minjoz University Hospital of Besançon. Viral RNA was extracted using the NucleoSpin^®^ RNA Virus or NucleoMag^®^ Pathogen kits (Macherey–Nagel, Hoerdt, France) according to the supplier’s recommendations and amplified by RT-PCR according to the protocols developed by the Berlin Charity Hospital (E gene) and the Pasteur Institute (RdRp gene) on LightCycler ^®^ 480 thermocycler (Roche, Boulogne-Billancourt, France). The positive controls used to obtain a calibration range were kindly provided by the Reference Centre for Respiratory Viruses (Institut Pasteur, Paris, France). The results were obtained in quantification cycle (cycle threshold, Ct) and transformed into log_10_ copies/mL using the calibration range.

In our study the persistence of a symptom was defined by the presence of at least one symptom related to SARS-CoV-2 infection at the time of the telephone survey and which could not be explained by another pathology, the patient was then included in the group with persistent symptoms (PS group). The resurgence of symptoms was defined as the appearance of at least one symptom after the acute phase of a SARS-CoV-2 infection that could not be explained by another pathology, the patient was then included in the group with symptom resurgence (group R). 

Our protocol has been validated by the clinical research unit of the NFCH.

Discrete variables were expressed as counts and percentages, continuous variables as mean, standard deviation and extreme, unless otherwise stated. Comparison between patients with or without persistent symptoms were performed using the Chi-squared test or exact Fisher test for qualitative variables and *t*-test or Wilcoxon test for quantitative data. All analyses were performed using SAS (version 9.4; SAS institute, Inc., Cary, NC, USA) with a significance level of 0.05.

## 3. Results

**Figure 1:**
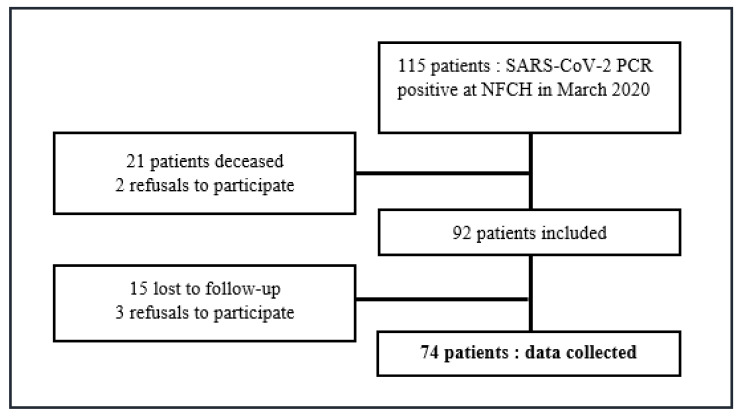
Flow Chart.

During the study period, 74 patients were included (Figure 1). The study population was 59.5% female and the mean age was 52.3 years (21–91.1) ± 18. Forty-seven point three percent were healthcare workers (HCWs). Fifty percent of the patients in the study had comorbidities, the most common of which were allergic condition, high blood pressure (HBP), VTED and diabetes. Patients responded to the telephone questionnaire on average 169.5 days (108–231) after the first symptoms appeared. A recrudescence of symptoms within 6 months of the onset was presented in 10/74 patients (13.51%) (Group R). 53/74 patients (71.62%, *n* = 53) had persistent symptoms, 6 months (±2) after the onset of symptoms (PS Group). Eight patients from group R were also included in group PS.

### 3.1. Description of the PS Group (n = 53)

**Figure 2:**
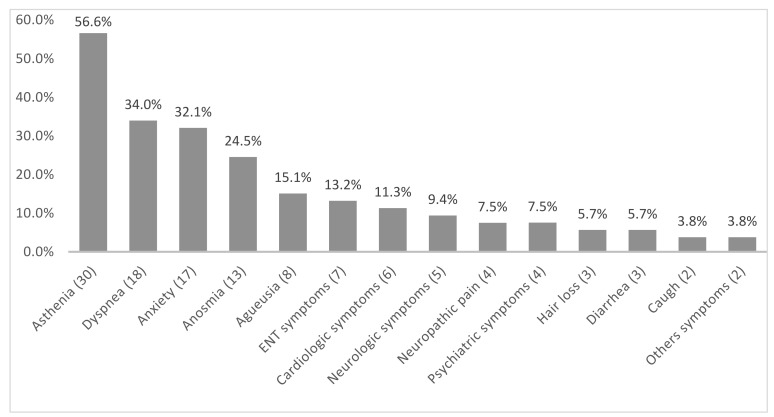
Proportion of persistent symptoms in the persistent symptom group (*n* = 53) after SARS-CoV-2 infection. ENT (ear, nose and throat) symptoms: nasal obstruction, nasal pain, rhinorrhea, nasal dryness, sneezing, odynophagia, dysphonia. Cardiologic symptoms: chest pain, palpitations, lipothymia. Neurologic symptoms: headache, dizziness, drowsiness. Neuropathic pain: myalgias, paresthesias of the limbs. Psychiatric symptoms: depressive syndrome, memory impairment, attention deficit. Other symptoms: epicondylitis, erectile dysfunction.

Of the 53 patients in the PS group, 56.6% were women. The average age was 54.7 years (21–89.2) ± 16.9. 23/74 patients (43.4%) were HCWs. Half of the patients described a single persistent symptom (45.2%, *n* = 24), others described two, three, four or more than five (respectively, 15.1% [*n* = 8], 17% [*n* = 9], 18.8% [*n* = 10], 3.8% [*n* = 2]). The most described symptoms were asthenia (56.6%, *n* = 30), dyspnea (34%, *n* = 18), anxiety (32.1% *n* = 17), anosmia (24.5%, *n* = 13) and agueusia (15.1% *n* = 8) (Figure 2). Fifty-six point six percent of patients (*n* = 30) had comorbidities and 45.3% (*n* = 24) required hospitalization (Table 1).

### 3.2. Comparison of the Two Groups: PS Group versus No-PS Group

There was no significant difference in gender and HCWs’ affiliation. Patients in the PS group were significantly older. 60.4% (*n* = 32) of patients in the PS group were older than 50 years old, compared with 19.1% (*n* = 4) in the no-PS group (*p* = 0.0048). Body mass index (BMI) was significantly higher in patients in the PS group (27.1 kg/m^2^ (17.9–42.4) ± 5.4 versus (vs) 24.3 kg/m^2^ (19.6–31.4) ± 3, *p* = 0.0071). Patients with comorbidities and chronic treatments were more present in the PS group with no statistically significant difference (respectively, 56.6% (*n* = 30) vs. 33.3 (*n* = 7) *p* = 0.0711 and 52.8% (*n* = 28) vs. 28.6 (*n* = 6) *p* = 0.0590). Patients in the PS group were significantly more hospitalized (45.3% (*n* = 24) vs. 19% (*n* = 4), *p* = 0.0359). Regarding tobacco consumption, the number of pack-years (PY) consumed was significantly higher in the PS group (4.8 PY (0–40) ± 9.3 vs. 1 PY (0–9) ± 2.2, *p* = 0.0074). Finally, in the PS group, odynophagia and nasal obstruction were less described in the inaugural symptoms of COVID-19 (respectively 28.3% (*n* = 15) vs. 57.1% (*n* = 12) *p* = 0.0202 and 18.9% (*n* = 10) vs. 42.9% (*n* = 9) *p* = 0.0332). The Ct of the E gene was significantly higher in the PS group (27.5 (15.2–39.8) ± 6.7 vs. 23.6 (13.5–34.3) ± 6.9). A low Ct corresponded to a high viral load (Table 1).

### 3.3. Comparison According to the Main Persistent Symptoms

#### 3.3.1. Asthenia

Persistent asthenia was described in 30 of 74 patients (40.5%) (Asth Group). In this group the patients were significantly older (59.2 years (29.1–89.2) ± 16.9 vs. 47.5 years (21–91.1) ± 17.3 *p* = 0.0045) and had a higher BMI (27.6 (17.9–38.6) ± 4.9 vs. 25.4 (19.1–42.4) ± 4.9 *p* = 0.0277). The Asth group also had more comorbidities (66.7%, (*n* = 20) vs. 38.6%, (*n* = 17) *p* = 0.0323) and in particular more HBP (33.3%, (*n* = 10) vs. 6.8%, (*n* = 3), *p* = 0.0048). Patients in the Asth group were more frequently hospitalized (*p* = 0.0003), oxygen therapy was more frequent (*p* = 0.0266) and the duration of hospitalization was longer (5.1 days (0–41) ± 8.9 vs. 2.5 (0–39) ± 7.3, *p* = 0.0008). These patients had more severe asthenia at the onset of the disease (*p* = 0.0158) and nasal obstruction was less present (6.7% (*n* = 2) vs. 38.6% (*n* = 17), *p* = 0.0024) in this group (Table 2a).

#### 3.3.2. Dyspnea 

Eighteen patients (24.3%) had persistent dyspnea (D Group). BMI was higher in D group compared to the no-D group. Indeed, 50% (*n* = 9) of patients in group D had a BMI > 30 kg/m^2^ compared to 16.1% (*n* = 9) of patients in no-D group (*p* = 0.0219). Allergic condition was significantly more represented in D group (47.1% (*n* = 8) vs. 14.3% (*n* = 8), *p* = 0.0077). Ten out of 18 patients were seen in consultation; eight patients were lost to follow-up. Four patients complained of dyspnea stage I (mMRC scale), three patients stage II, two patients stage III and 1patient stage V. All of these patients had normal auscultation and oxygen saturation at the time of consultation. One patient out of the 10 had desaturation during 6WT at 87% as well as hypoxia on blood gas at 71.6 mmHg. Four out of ten patients had an abnormality on the LFT, two probably had underlying asthmatic disease, one patient had chest distention and one patient had possible sequelae of infection in the absence of pre-existing lung damage. All chest CT scans performed showed no or minimal involvement typical of COVID-19 (<10%) (Table 2b).

#### 3.3.3. Anxiety

Seventeen patients (23%) had sequelae of anxiety (Anx group). Patients in the Anx group were older (*p* = 0.0197) and had more comorbidity (88.2% (*n* = 15) vs. 38.6% (*n* = 22), *p* = 0.0006). In the Anx group, patients had more severe anxiety and asthenia at baseline (*p* < 0.0001 and *p* = 0.0485, respectively) (Table 2c).

#### 3.3.4. Anosmia

Thirteen patients (17.6%) had persistent anosmia (An Group). In An group, patients had more frequent facial headaches, abdominal pain and nausea at the onset of infection, compared to the no-An group (respectively, 69.2% (*n* = 9) vs. 29.5% (*n* = 18) *p* = 0.0108, 53.8% (*n* = 7) vs. 18% (*n* = 11) *p* = 0.0117 and 69.2% (*n* = 9) vs. 34.4% (*n* = 21) *p* = 0.0294). Patients in the An group were less likely to receive oxygen therapy (Table 2d).

#### 3.3.5. Agueusia

Eight patients (10.8%) had persistent agueusia (Ag group). In the Ag group, women were significantly more represented (100% (*n* = 8) vs. 54.6% (*n* = 36), *p* = 0.0181). Patients in the Ag group had more frequent facial headaches at the beginning of the disease compared to the no-Ag group (75% (*n* = 6) vs. 31.8% (*n* = 21), *p* = 0.0450).

### 3.4. Description of Patients with Resurgence of Symptoms (n = 10) 

In group R, six out of ten patients were female and the mean age was 51.6 years (29.1–81.7) ± 17.1. Symptom resurgence occurred on average 103.3 days (21–195) after the onset of the first symptoms. The most frequently presented symptoms were asthenia, chest pain, cough and dysgeusia (*n* = 8, *n* = 5, *n* = 4, *n* = 4, respectively). No patient required hospitalization during the resurgence of symptoms. Two out of ten patients with a recrudescence of symptoms gave a second PCR sample, which was negative (Table 3).

### 3.5. Comparison of the Two Groups: R Group vs. No-R Group

In R group, HCWs were more represented (80%, (*n* = 8) vs. 42.2% (*n* = 27), *p* = 0.0396). Patients in the R group more frequently presented facial headaches at the onset of the disease (*p* = 0.0309). The time to medical consultation after the onset of the first symptoms of COVID-19 was shorter in the R group (2.2 days (1–5) ± 1.4 vs. 3.8 days (1–15) ± 3.2, *p* = 0.0141) (Table 3).

## 4. Discussion

In our sample population, the mean age was 52.3 years old, which is similar to the general population affected by COVID-19 [11,12,13]. Some studies show a younger age but included the minor patients that we excluded in COVEVOL [8,11]. 

HCWs were widely represented in the study (47.3%). In the first wave, easier access to the test for HCWs allowed their inclusion and may explain this proportion. The percentage of women was higher in our study (59.5%) compared to the literature [12,13,14]. This difference is explained by the fact that the majority of the HCWs patients were women (15) and were highly represented in our study. The presence of comorbidities was also higher in our study (50%) [4,11,15]. Allergic condition was the most represented comorbidity. The presence of an allergic condition in the comorbidities may have overestimated the number of comorbidities. Other comorbidities were similar to those in the literature [4,11].

In our study, 71% of the patients had persistent symptoms 6 months after a COVID-19 infection. The study by Peluso and al. of 179 patients showed a similar frequency 12 to 20 weeks after the onset of infection [16]. A Chinese study of 1733 patients showed that 76% of patients reported at least one symptom at 6 months’ follow-up [12]. Some studies found a higher rate of persistent symptoms but only looked at hospitalized patients [17,18]. On the opposite, other studies found a lower rate but only included patients treated as outpatient [14,19,20]. Moreno-Pérez and al. found that 50.9% of patients had at least one symptom at 3 months but they did not take into account psychological symptoms that may have underestimated their results [21]. Regarding the nature of persistent symptoms, the two most frequent were asthenia and dyspnea according to the literature [12,15,16,17,18,19,20,21,22,23,24,25]. The frequency of anxiety, anosmia, agueusia, ENT, cardiological, neurological, painful, psychiatric symptoms were similar to other studies [11,12,13,15,19,20].

In our study we separated age and BMI from other comorbidities. Patients in the PS group had a higher BMI (27.1 kg/m^2^ on average) and were older (60.4% were over 50 years old) in accordance with other studies [19,26,27]. Other comorbidities and chronic medication appeared to be more present in the PS group but without significant difference. In the literature, comorbidities are a significant risk for persistence of symptoms [19,22,26]. The inclusion of BMI and age in the comorbidities of our study would have potentially led to similar results.

It is currently accepted that advanced age, obesity and comorbidities are risk factors for severe COVID-19 infection [3,26,28]. In COVEVOL, hospitalized patients with more severe disease were more present in the PS group. Tenforde et al. reported that risk factors for severe COVID-19 infection were also risk factors for not regaining one’s previous state of health [19]. Other studies corroborate these findings [14] supporting our hypothesis that patients with severe COVID-19 disease were at higher risk for persistent symptoms. However, we did not find similar results in patients hospitalized in intensive care unit (ICU), unlike the study of Huang and al. [12], probably due to a lack of statistical power of the too small number of patients. The relationship between the severity of COVID-19 and the increased likelihood of persistent symptoms may be explained by the immune response to SARS-CoV-2 virus that stimulates the production of cytokines and other inflammatory mediators, with higher concentrations found in patients with a more severe clinical form [11]. The hypothesis of the prolonged persistence of low-grade inflammation is favored by some authors and is consistent with the pathophysiology of severe COVID-19 [29]. Odynophagia and nasal obstruction were less present in patients in the PS group. In the literature, symptoms suggestive of mild viral pathology were less present in the acute phase in patients with severe COVID-19 [30]. We can therefore assume that they may be predictive of the absence of persistent symptoms at a distance from the disease as our results may suggest. Regarding smoking, the number of pack-years consumed was higher in the PS group. A meta-analysis including 47 studies showed that a history of smoking was associated with an increased risk of severe disease [31]. It can therefore be assumed that active or former smoking may also be a risk factor for persistence of symptoms since it promotes initial severe disease involvement. Further studies are needed to explore this hypothesis. The viral load in patients in the PS group was significantly lower (E gene Ct: 27.5 log_10_ copies/mL). Our results are contradictory with the literature. Indeed, Klement-Frutos et al. showed that the viral load was higher in deceased patients compared to survivors and that a low viral load was associated with a better prognosis [32]. Our previous hypotheses suggested that patients in the PS group had a higher viral load because they tended to have a more severe form. However, our study has an important limitation as patients did not perform their tests at the same time. Seventy-one percent of the patients in the no-PS group performed PCR testing within the first three days after the onset of their symptoms compared to only 15% of the patients in the PS group. Studies have shown that the viral load peaks 1.1 days before the onset of symptoms and then gradually decreases [33]. Our hypothesis is that the viral load of the patients in the PS group had probably already decreased at the time of testing, having performed the test later, compared to the no-PS group. It would have been preferable to test all patients at the same time, i.e., the same number of days after the onset of the first symptoms, in order to reliably interpret these results. Finally, we did not find a significant difference in gender in accordance with other studies [21].

Persistent asthenia was present in 40.5% of the patients in our study after 6 months of follow-up. These patients were older (mean 59.2 years), had a higher BMI (on average 27.6 kg/m^2^) and 66.7% had at least one comorbidity including HBP in 33.3%. The risk factors highlighted in the persistence of asthenia are the same as those found in the overall persistence of symptoms and were correlated with the risk of severe COVID-19. Patients with persistent asthenia were hospitalized more often, had received more oxygen therapy, and their hospitalization was more prolonged. All of this suggests a more severe SARS-CoV-2 infection. In the literature, older subjects generally have more severe forms of infection and their lower ability to recover from acute infection compared with younger patients corroborate these findings [19,27]. It would have been interesting to eliminate factors predisposing to asthenia (anemia, hypothyroidism, vitamin D deficiency, etc.) as some studies point out [34,35]. The prevalence of asthenia is in line with other SARS epidemics in which a large proportion of patients were diagnosed with myalgic encephalomyelitis syndrome and chronic fatigue syndrome [36].

Persistent dyspnea was present in 18 patients (24.3%) (D Group). These patients had a higher BMI (50% had a BMI > 30 kg/m^2^) in accordance with the literature [22]. Patients with obesity generally have impaired ventilatory capacity. Studies have shown that a high BMI is associated with an increased risk of ARDS and therefore mortality in COVID-19 disease [4]. Indeed, obesity is frequently associated with pulmonary pathologies, such as sleep apnea syndrome, asthma and restrictive syndrome. Obesity is also associated with changes in ventilatory mechanics, a decrease of respiratory muscle strength and a reduction in effective lung volumes that alter pulmonary gas exchange [37]. This phenomenon may explain why these patients are at a higher risk of persistent dyspnea at a distance from the infection. COVEVOL found the presence of an allergic condition more frequently in D group. Several studies have shown that asthma and allergic diseases are not risk factors for COVID and in particular for severe COVID [4,12]. One study [27] showed that asthma was a risk factor for prolonged symptoms at 28 days but not for longer-term persistent symptoms. The possible link between allergic condition and persistent dyspnea requires further investigation. Ten out of the 18 patients in D Group were re-examined in consultation. Only one patient had a desaturation at 6WT of 87% and a hypoxia of 71.6 mmHg at blood gas. In a Chinese study [12], the median walking distance at 6WT was below normal reference values in about a quarter of patients at 6 months, a prevalence similar to that observed in SARS and MERS survivors [38]. All patients seen in consultation had normal auscultation and oxygen saturation and chest CT scan showed no or minimal abnormality (<10%). Another study showed similar results [20]. McGroder et al. showed that there was no correlation between persistent dyspnea and chest CT scan abnormalities at 4-month follow-up [39]. Similarly, in this study, abnormalities on the 6WT were correlated with the sensation of dyspnea and not with radiological abnormalities, dyspnea appeared to be associated with physical deconditioning and a higher fragility score [39]. In our study, patients with an abnormal LFT were likely to have pre-existing respiratory diseases (asthma, chest distention). Only one patient presented LFT results in favor of possible sequelae of infection provided there was no underlying pre-existing abnormality. It would have been interesting to exclude, according to current recommendations [40], heart failure or anemia, and to consider measuring the Nimjegen score in the absence of objective abnormalities in order not to omit a hyperventilation syndrome. 

Persistent anxiety after COVID-19 infection is frequently described in the literature [16,20]. In our study 17 patients (23%) had persistent anxiety 6 months after infection. These patients had more comorbidities (88.2% had at least one comorbidity) and were older (58.8% were over 60 years old). Advanced age and the presence of comorbidities are risk factors for severe infection and mortality in COVID-19 [3,26,28]. This information relayed by the media may have contributed to the occurrence of anxiety in this population. A Chinese study of 1210 patients during the COVID-19 pandemic showed that poor self-rated health by the patient was significantly associated with higher levels of stress, anxiety and depression [41]. Some studies have shown that epidemic diseases have psychological consequences in both infected and uninfected individuals [13]. It would have been interesting to compare our study population with a control population in order to conclude the imputability between COVID-19 infection and psychological damage during this pandemic. The group of patients with persistent anxiety felt more anxious at the beginning of the disease (47% “highly anxious” or “very highly anxious”). A study of 32,000 patients in the United States suggests a causality between pre-existing psychological distress and persistent mental, emotional and behavioral symptoms of “long COVID” [42]. Patients with persistent anxiety had greater asthenia in the acute phase (64.7% WHO performance status >2). Huang et al. showed that in hospitalized patients, the persistence of anxiety disorders was correlated with the severity of the disease at the initial phase [12].

Persistent anosmia was described in 13/74 patients (17.6%). A study by Lechien et al. of 1363 patients showed that 4.7% had persistent anosmia or hyposmia at 6 months [43]. Other studies corroborate these results [29]. As we did not use an objective test to measure olfaction in COVEVOL, our result may have been overestimated. Patients in the An Group had significantly less benefited from oxygen therapy than patients in no-An group (0 vs. 27.9%; *p* = 0.0312). According to several studies, anosmia at the time of SARS-CoV-2 infection tends to affect young, female patients and is associated with a favorable prognosis [30,43]. The difference in the use of oxygen therapy between the An and no-An groups may be explained by the greater severity of COVID-19 expected in patients without ENT disease. In the An group, facial headaches were significantly more present in the acute phase of COVID-19 disease. Eliezer et al. showed by magnetic resonance imaging that agueusia/anosmia could result from bilateral inflammatory obstruction of the olfactory clefts caused by SARS-CoV-2 infection, which may explain that in our study patients with sinusitis-like symptoms at baseline had persistent anosmia/agueusia [44]. We find the same results in patients with persistent agueusia. In COVEVOL, patients in the An group had more frequently presented nausea and abdominal pain at the beginning of the disease. According to a study, gastrointestinal symptoms were rather predictive of a severe form of COVID-19 disease [45]. Our discordant results with the literature may be explained by the small size in the An group.

In addition, persistent agueusia at 6 months was present in 8/74 patients (10.8%). In the initial phase of COVID-19 disease, 47 patients had agueusia. A study by Chiesa-Estomba showed that 90.6% of patients who reported a loss of taste appeared to have regained taste two months after the end of the coronavirus disease [46]. We find similar results. Our study showed that women were significantly more present in the Ag group compared to the no-Ag group (100% [*n* = 8] vs. 54.6% [*n* = 36], *p* = 0.0181). However, the data in the literature do not corroborate this result, so it is difficult to assume that female gender is predictive of persistent agueusia given the small size of our study.

The cases of symptom recrudescence found in our study were few (10/74) and did not require hospitalization. The main symptoms reported were asthenia, chest pain, cough and dysgeusia (*n* = 8, *n* = 5, *n* = 4, *n* = 4, respectively). However, the cases of resurgence of symptoms described in the literature remain rare and present very variable clinical characteristics, ranging from simple flu-like syndrome [47,48] to ARDS leading to death [49]. In the literature the resurgence seems to be related to viral reactivation [47,48,49,50]. In COVEVOL it was based on clinical data, by self-reported symptoms, and not virological data. Indeed, viral reactivation could not be proven by systematically taking a new sample at the time of the reappearance of symptoms. Only two patients performed an RT-PCR test, two days after the resurgence of the symptoms for the first one, and a month later for the second one, both were negative. These results do not allow us to conclude that there is a link between a resurgence of symptoms and viral reactivation; regular monitoring of the viral load in these patients could have helped us understand this phenomenon. In our study, HCWs were more represented in the R group. ANOSVID, another study conducted in our institution [51], found similar results and suggested that recrudescent or persistent symptoms could be favored by post-traumatic stress or due to stress generated by the responsibility of care in the epidemic context. However, there is currently no description in the literature of specific clinical or demographic characteristics that could predict the resurgence of symptoms. Of the patients in group R, 3/10 were hospitalized in the conventional sector in the acute phase of the disease, none in the ICU and one patient received oxygen therapy. We found no significant association between initial symptom severity and symptom recrudescence similar to what has been described for repeated infections with other endemic coronaviruses [52]. The time to first consultation in R group was significantly shorter (2.2 days vs. 3.8 days). This phenomenon can be explained by the fact that R group is mostly represented by HCWs (8/10 patients). The daily contact with seriously ill patients during a worldwide pandemic may have favored the onset of stress and, consequently, possibly somatization, which may explain easy anxiety and rapid consultation.

In COVEVOL, selection bias was limited by systematically including all patients who presented a positive COVID-19 PCR test during March 2020 at NFCH. In our study, extrapolation to the general population is difficult due to the small sample size, however our population is representative of the population affected by COVID in the general population which increases the external validity of the study. The bias related to the loss of follow-up was limited by calling the patients twice and leaving a voice message on their answering machine. The telephone questionnaire was completed by a single interviewer to limit subjective evaluation bias. There is a systematic bias related to the lack of memorization of the respondents, our study being based on a declarative collection mode. This bias was limited by using the data available in COVIDES and in the patient digital record for comorbidities and initial symptoms. Persistent symptoms “asthenia, dyspnea, anxiety, anosmia and agueusia” may have been overestimated compared with other symptoms being routinely asked during the standardized telephone interview while other symptoms were self-reported. Let’s take the example of anxiety, which is highly represented in our study, while depression is exceptional. If all symptoms had been self-reported, the study might not have found as much difference in frequency between the two symptoms. Baseline data for LFT and 6WT are not available to compare our data.

## 5. Conclusions

Persistent symptoms 6 months after SARS-CoV-2 infection are common. High age, high BMI and hospitalization appear to be predictive of persistent symptoms. Conversely, ENT forms in the acute phase of COVID-19 disease appear to be predictive of the absence of prolonged symptoms. Further large-scale studies are needed to extrapolate our results. The identification of favorable factors is necessary for the understanding of this phenomenon and an adapted management.

## Figures and Tables

**Table 1:** Demographic and clinical characteristics in 74 COVID-19 patients with or without persistent symptoms after SARS-CoV-2 infection, Nord Franche-Comte Hospital, France.

Persistent Symptoms		No-PS Group (*n* = 21) (28.4%)	PS Group (*n* = 53) (71.6%)	Total (*n* = 74) (100%)	*p*-(Value)
Demographic and baseline characteristics					
Age (years) (mean, extremes, SD)		46.1 (23.7–91.1) ± 19.6	54.7 (21–89.2) ± 16.9	52.3 (21–91.1) ± 18	0.0625
(number, %)	(18–30)	4 (19.1)	5 (9.4)	9 (12.2)	0.0048
	(31–40)	7 (33.3)	8 (15.1)	15 (20.3)	
	(41–50)	6 (28.6)	8 (15.1)	14 (18.9)	
	(51–60)	0	9 (17)	9 (12.2)	
	(61–70)	0	14 (26.4)	14 (18.9)	
	>71	4 (19.1)	9 (17)	13 (17.5)	
Sex (number, %)	Female	14 (66.7)	30 (56.6)	44 (59.5)	0.4267
HCWs (number, %)		12 (57.1)	23 (43.4)	35 (47.3)	0.2856
BMI (kg/m^2^) (mean, extremes, SD)		24.3 (19.6–31.4) ± 3	27.1 (17.9–42.4) ± 5.4	26.3 (17.9–42.4) ± 5	0.0071
(number, %)	<25	13 (61.9)	25 (47.2)	38 (51.4)	0.0350
	(25–30)	7 (33.3)	11 (20.7)	18 (24.3)	
	>30	1 (4.8)	17 (32.1)	18 (24.3)	
*Tobacco (number, %)*	Current smoking	0	4 (7.5)	4 (5.4)	0.5725
	Former smoker	5 (23.8)	15 (28.3)	20 (27)	0.6948
Number of pack–year *(mean, extremes, SD)*		1 (0–9) ± 2.2	4.8 (0–40) ± 9.3	3.7 (0–40) ± 8.1	0.0074
*Alcohol (number, %)*	No alcohol consumption	4 (19)	12 (22.6)	16 (21.6)	1
	Occasional drinking	14 (66.7)	34 (64.2)	48 (64.9)	
	Daily alcohol consumption	3 (14.3)	7 (13.2)	10 (13.5)	
*Comorbidities (number, %)*		7 (33.3)	30 (56.6)	37 (50)	0.0711
Allergic condition		5 (23.8)	11 (21.1)	16 (21.9)	0.7657
High blood pressure		2 (9.5)	11 (20.7)	13 (17.6)	0.3264
Venous thromboembolic disease		0	7 (13.2)	7 (9.5)	0.1808
Diabetes		1 (4.8)	5 (9.4)	6 (8.1)	0.6681
Asthma		2 (9.5)	3 (5.7)	5 (6.8)	0.6163
Heart failure		2 (9.5)	1 (1.9)	3 (4)	0.1922
Active malignancy		0	3 (5.7)	3 (4)	0.5536
Obstructive sleep apnea syndrome		1 (4.8)	2 (3.8)	3 (4)	1
Coronary artery disease		1 (4.8)	5 (9.4)	2 (2.7)	0.4954
Autoimmune condition		2 (3.8)	0	2 (2.7)	1
Chronic obstructive pulmonary disease		0	1 (1.9)	1 (1.3)	1
Chronic kidney failure		0	1 (1.9)	1 (1.3)	1
Malignancy in remission		0	1 (1.9)	1 (1.3)	1
Charlson score	0	17 (81)	35 (66)	52 (70.3)	0.2658
	(1–10)	4 (19)	18 (34)	22 (29.7)	
*Chronic treatment* (number, %)		6 (28.6)	28 (52.8)	34 (46)	0.0590
	CEIs	2 (9.5)	4 (7.5)	6 (8.1)	1
	Statins	1 (4.8)	5 (9.4)	6 (8.1)	0.6681
	Oral antidiabetics	1 (4.8)	4 (7.5)	5 (6.8)	1
	NSAIDs	0	4 (7.5)	4 (5.4)	0.5725
	ARBs	1 (4.8)	1 (1.9)	2 (2.7)	0.4898
	Corticosteroids	0	1 (1.9)	1 (1.3)	1
	Immunosuppressor	1 (1.9)	0	1 (1.3)	1
Clinical characteristics					
*Initial symptomatology*					
General symptoms (number, %)	Asthenia	21 (100)	48 (90.6)	69 (93.2)	0.3129
	Headaches	17 (80.9)	41 (77.4)	58 (78.4)	1
	Fever	14 (66.7)	42 (79.2)	56 (75.7)	0.2555
	Myalgia	14 (66.7)	39 (73.6)	53 (71.6)	0.5518
ENT symptoms (number, %)	Dysgueusia	14 (66.7)	33 (62.3)	47 (63.5)	0.7228
	Anosmia	11 (52.4)	34 (64.2)	45 (60.8)	0.3498
	Rhinorrhea	9 (42.9)	25 (47.2)	34 (46)	0.7372
	Facial headaches	6 (28.6)	21 (39.6)	27 (36.5)	0.3733
	Odynophagy	12 (57.1)	15 (28.3)	27 (36.5)	0.0202
	Sneezing	8 (38.1)	17 (32.1)	25 (33.8)	0.6216
	Nasal obstruction	9 (42.9)	10 (18.9)	19 (25.7)	0.0332
	Tinnitus	2 (9.5)	6 (11.3)	8 (10.8)	1
	Hypoacusis	1 (4.8)	4 (7.5)	5 (6.8)	1
	Epistaxis	1 (4.8)	3 (5.7)	4 (5.4)	1
Cardiopulmonary symptoms (number, %)	Cough	18 (85.7)	43 (81.1)	61 (82.4)	0.7466
	Dyspnea	4 (19)	21 (39.6)	25 (33.8)	0.0916
	Chest pain	5 (23.8)	15 (28.3)	20 (27)	0.6948
	Hemoptysis	0	3 (5.7)	3 (4)	0.5536
Digestive symptoms (number, %)	Diarrhea	10 (47.6)	30 (56.6)	40 (54)	0.4844
	Nausea	7 (33.3)	23 (43.4)	30 (40.5)	0.4267
	Abdominal pain	4 (19)	14 (26.4)	18 (24.3)	0.5054
	Vomiting	2 (9.5)	4 (7.5)	6 (8.1)	1
Anxiety (number, %)	0	15 (71.4)	26 (49.1)	41 (55.5)	0.0585
	(1,2)	6 (28.6)	17 (32.1)	23 (31.1)	
	(3,4)	0	10 (18.9)	10 (13.5)	
Duration of symptoms of SARS–CoV–2 infection (days) (mean, extremes, SD)		12.4 (3–28) ± 6.7	14.3 (3–51) ± 9.4	13.7 (3–51) ± 8.7	0.4170
Hospitalization (number, %)		4 (19)	24 (45.3)	28 (37.8)	0.0359
Conventional hospitalization		4 (19)	23 (43.4)	27 (36.5)	0.0498
Intensive care unit		0	4 (7.5)	4 (5.4)	0.5725
Duration of hospitalization (days) (mean, extremes, SD)		2.0 (0–21) ± 5.9	4.1 (0–41) ± 8.7	3.5 (0–41) ± 8	0.3184
Oxygenotherapy (number, %)		2 (9.5)	15 (28.3)	17 (23)	0.1256
Mechanical ventilation (number, %)		0	3 (5.7)	3 (4)	0.5536
Ct gene E (log_10_ copies/mL) (mean, extremes, SD)		23.6 (13.5–34.3) ± 6.9	27.5 (15.2–39.8) ± 6.7	26.4 (13.5–39.8) ± 6.9	0.0303

HCWs: healthcare workers; BMI: body mass index; ENT symptoms: ear, nose and throat symptoms; CEIs: cholinesterase inhibitor; NSAIDs: nonsteroidal anti-inflammatory drugs; ARBs: angiotension II receptor blockers; anxiety scale: 0: not anxious, 1: slightly anxious, 2: moderately anxious, 3: highly anxious, 4: very highly anxious.

**Table 2:** Comparison of patients by most common persistent symptoms.

2a. Asthenia			No-Asth Group (*n* = 44) (59.5%)	Asth Group (*n* = 30) (40.5%)	Total (*n* = 74) (100%)	*p*-(Value)
Demographic and baseline characteristics						
Age (mean, extremes, SD)			47.5 (21–91.1) ± 17.3	59.2 (29.1–89.2) ± 16.9	52.3 (21–91.1) ± 18	0.0045
Sex (number, %)	Female		27 (61.4)	17 (56.7)	44 (59.5)	0.8102
HCWs (number, %)			25 (56.8)	10 (33.3)	35 (47.3)	0.0597
BMI (kg/m^2^) (mean, extremes, SD)			25.4 (19.1–42.4) ± 4.9	27.6 (17.9–38.6) ± 4.9	26.3 (17.9–42.4) ± 5	0.0277
Comorbidities (number, %)			17 (38.6)	20 (66.7)	37 (50)	0.0323
	High blood pressure		3 (6.8)	10 (33.3)	13 (17.6)	0.0048
Charlson score	0		35 (79.6)	17 (56.7)	52 (70.3)	0.0418
	(1–10)		9 (20.4)	13 (43.3)	22 (29.7)	
Chronic treatment (number, %)			16 (36.4)	18 (60)	34 (46)	0.0588
Clinical characteristics						
Initial symptomatology						
Asthenia	(0–2)		31 (70.5)	12 (40)	43 (58.1)	0.0158
	(3–4)		13 (29.5)	18 (60)	31 (41.9)	
Nasal obstruction			17 (38.6)	2 (6.7)	19 (25.7)	0.0024
Hospitalization (number, %)			9 (20.4)	19 (63.3)	28 (37.8)	0.0003
	Conventional hospitalization		9 (20.4)	18 (60)	27 (36.5)	0.0012
	Intensive care unit		2 (4.5)	2 (6.7)	4 (5.4)	0.5725
					
Duration of hospitalization (days) (mean, extremes, SD)			2.5 (0–39) ± 7.3	5.1 (0–41) ± 8.9	3.5 (0–41) ± 8	0.0008
					
Oxygenotherapy (number, %)			6 (13.6)	11 (36.7)	17 (23)	0.0266
Mechanical ventilation (number, %)			1 (2.3)	2 (6.7)	3 (4)	0.5622
Ct gene E (log_10_ copies/mL) (mean, extremes, SD)			25 (13.5–39.8) ± 7.3	28.5 (16.8–38.9) ± 5.9	26.4 (13.5–39.8) ± 6.9	0.0436
**2b. Dyspnea**			**no-D Group** **(*n* = 56)** **(75,7%)**	**D Group (*n* = 18)** **(24.3%)**	**Total (*n* = 74)** **(100%)**	***p*** **-(Value)**
Demographic and baseline characteristics						
Age (mean, extremes, SD)			52.9 (23.7–91.1) ± 17.9	50.3 (21–89.2) ± 18.5	52.3 (21–91.1) ± 18	0.6291
Sex (number, %)	Female		33 (58.9)	11 (61.1)	44 (59.5)	1
HCWs (number, %)			27 (48.2)	8 (44.4)	35 (47.3)	1
BMI (kg/m^2^) (mean, extremes, SD)			25.6 (17.9–42.4) ± 4.8	28.2 (21–38.6) ± 5.1	26.3 (17.9–42.4) ± 5	0.0524
(number, %)	<25		32 (57.1))	6 (33.3)	38 (51.4)	0.0219
	(25–30)		15 (26.8)	3 (16.7)	18 (24.3)	
	>30		9 (16.1)	9 (50)	18 (24.3)	
Comorbidities (number, %)			27 (48.2)	10 (55.6)	37 (50)	0.7870
	Allergic condition		8 (14.3)	8 (47.1)	16 (21.9)	0.0077
Charlson score	0		37 (66.1)	15 (83.3)	52 (70.3)	0.2381
	(1–10)		19 (33.9)	3 (16.7)	22 (29.7)	
Clinical characteristics						
Hospitalization (number, %)			18 (32.1)	10 (55.6)	28 (37.8)	0.0966
Conventional hospitalization			17 (30.4)	10 (55.6)	27 (36.5)	0.0894
Intensive care unit			3 (5.4)	1 (5.6)	4 (5.4)	1
**2c. Anxiety** **HCWs: Healthcare Worker** **BMI: Body Mass Index**			**no-Anx Group** **(*n* = 57)** **(77%)**	**Anx Group (*n* = 17)** **(23%)**	**Total (*n* = 74)** **(100%)**	***p*** **-(Value)**
Demographic and baseline characteristics						
Age (mean, extremes, SD)			49.7 [22.4–91.1] ±17.3	60.9[21–89.2] ±18.1	52.3 [21–91.1] ±18	0.0197
(Number, %)	(18–30)		7 (12.3)	2 (11.8)	9 (12.2)	0.0462
	(31–40)		15 (26.3)	0	15 (20.3)	
	(41–50)		12 (21)	2 (11.8)	14 (18.9)	
	(51–60)		6 (10.5)	3 (17.6)	9 (12.2)	
	(61–70)		10(17.5)	4 (23.5)	14 (18.9)	
	>71		7(12.3)	6 (35.3)	13 (17.5)	
Sex (number, %)	Female		34 (59.7)	10 (58.8)	44 (59.5)	1
HCWs (number, %)			28 (49.1)	7 (41.2)	35 (47.3)	0.5934
BMI (kg/m^2^) (mean, extremes, SD)			25.8 (17.9–42.4) ± 4.8	27.7 (19.6–39.1) ± 5.5	26.3 (17.9–42.4) ± 5	0.2296
Comorbidities (number, %)			22 (38.6)	15 (88.2)	37 (50)	0.0006
Charlson score	0		46 (80.7)	6 (35.3)	52 (70.3)	0.0007
	(1–10)		11 (19.3)	11 (64.7)	22 (29.7)	
Clinical characteristics						
Initial symptomatology						
Asthenia	(0–2)		37 (64.9)	6 (35.3)	43 (58.1)	0.0485
	(3–4)		20 (35.1)	11 (64.7)	31 (41.9)	
Anxiety (number, %)	0		34 (59.7)	7 (41.2)	41 (55.5)	<0.0001
	(1–2)		21 (36.8)	2 (11.8)	23 (31.1)	
	(3–4)		2 (3.5)	8 (47)	10 (13.5)	
Hospitalization (number, %)			19 (33.3)	9 (52.9)	28 (37.8)	0.1640
**2d. Anosmia**			**no-An Group** **(*n* = 61)** **(82.4%)**	**An Group (*n* = 13)** **(17.6%)**	**Total (*n* = 74)** **(100%)**	***p*** **-(Value)**
Demographic and baseline characteristics						
Age (mean, extremes, SD)			53.5 (22.4–91.1) ± 18.2	46.3 (21–67.3) ± 16.4	52.3 (21–91.1) ± 18	0.2052
Sex (number, %)		Female	36 (59)	8 (61.5)	44 (59.5)	1
BMI (kg/m^2^) (mean, extremes, SD)			26.6 (17.9–42.4) ± 5.1	24.9 (19.6–32.5) ± 4	26.3 (17.9–42.4) ± 5	0.3663
Comorbidities (number, %)			31 (50.8)	6 (46.1)	37 (50)	1
Charlson score		0	40 (65.6)	12 (92.3)	52 (70.3)	0.0920
		(1–10)	21 (34.4)	1(7.7)	22 (29.7)	
Clinical characteristics						
Initial symptomatology						
Facial headaches (number, %)			18 (29.5)	9 (69.2)	27 (36.5)	0.0108
Nausea (number, %)			21 (34.4)	9 (69.2)	30 (40.5)	0.0294
Abdominal pain (number, %)			11 (18)	7 (53.8)	18 (24.3)	0.0117
Number of initial symptoms		(0–5)	6 (9.8)	0	6 (8.1)	0.0594
		(6–10)	22 (36.1)	1 (7.7)	23 (31)	
		(11–15)	25 (41)	10 (76.9)	35 (47.3)	
		(16–20)	8 (13.1)	2 (15.4)	10 (13.6)	
Hospitalization (number, %)			25 (41)	3 (23.1)	28 (37.8)	0.3467
Oxygenotherapy (number, %)			17 (27.9)	0	17 (23)	0.0312

HCWs: healthcare workers; BMI: body mass index; asthenia measured according to the WHO performance status, where 0: fully active, able to carry on all predisease performance without restriction; 1: restricted in physically strenuous activity but ambulatory and able to carry out work of a light or sedentary nature, e.g., light house work, office work; 2: ambulatory and capable of all self-care but unable to carry out any work activities and up and about more than 50% of waking hours; 3: capable of only limited self-care, confined to bed or chair more than 50% of waking hours; 4: completely disabled, cannot carry on any self-care, totally confined to bed or chair. Anxiety scale: 0: not anxious, 1: slightly anxious, 2: moderately anxious, 3: highly anxious, 4: very highly anxious.

**Table 3:** Demographic and clinical characteristics in 74 COVID-19 patients with or without resurgence symptoms after SARS-CoV-2 infection, Nord Franche-Comte Hospital, France.

Resurgence Symptoms		No-R Group (*n* = 64) (86.5%)	R Group (*n* = 10) (13.5%)	Total (*n* = 74) (100%)	*p*-(Value)
Demographic and baseline characteristics					
Age (mean, extremes, SD) (number, %)		52.3 (21–91.1) ± 18.2	51.6 (29.1–81.7) ± 17.1	52.3 (21–91.1) ± 18	0.9069
	(18–30)	8 (12.5)	1 (10.0)	9 (12.2)	0.9868
	(31–40)	13 (20.3)	2 (20.0)	15 (20.3)	
	(41–50)	11 (17.2)	3 (30.0)	14 (18.9)	
	(51–60)	8 (12.5)	1 (10.0)	9 (12.2)	
	(61–70)	12 (18.8)	2 (20.0)	14 (18.9)	
	>71	12 (18.8)	1 (10.0)	13 (17.5)	
Sex (number, %)	Female	38 (59.4)	6 (60)	44 (59.5)	1
HCWs (number, %)	No	27 (42.2)	8 (80)	35 (47.3)	0.0396
BMI (kg/m^2^)	<25	32 (50)	6 (60)	38 (51.4)	1
	(25–30)	16 (25)	2 (20)	18 (24.3)	
	>30	16 (25)	2 (20)	18 (24.3)	
Comorbidities (number, %)		32 (50)	5 (50)	37 (50)	1
Clinical characteristics and outcome					
Duration of symptoms of SARS-CoV-2 infection (days) (mean, extremes, SD)		14 (3–51) ± 9.1	12.4 (3–21) ± 6.1	13.7 (3–51) ± 8.7	0.6030
Hospitalization		25 (39.1)	3 (30)	28 (37.8)	0.7330
Duration of hospitalization (days) (mean, extremes, SD)		3.9 (0–41) ± 8.5	3.8 (0–9) ± 12	3.5 (0–41) ± 8	0.0903
Initial symptomatology					
Facial headaches		20 (31.3)	7 (70)	27 (36.5)	0.0309
Anxiety		26 (40.6)	7 (70)	33 (44.6)	0.0992
Time from symptom onset to first visit (days) (mean, extremes, SD)		3.8 (1–15) ± 3.2	2.2 (1–5) ± 1.4	3.6 (1–15) ± 3.1	0.0141

HCWs: healthcare workers; BMI: body mass index.

## Data Availability

All data generated or analysed during this study are available at the clinical research unit, Hôpital Nord Franche- Comté, Belfort, France.
